# T4-Related Bacteriophage LIMEstone Isolates for the Control of Soft Rot on Potato Caused by ‘Dickeya solani’

**DOI:** 10.1371/journal.pone.0033227

**Published:** 2012-03-07

**Authors:** Evelien M. Adriaenssens, Johan Van Vaerenbergh, Dieter Vandenheuvel, Vincent Dunon, Pieter-Jan Ceyssens, Maurice De Proft, Andrew M. Kropinski, Jean-Paul Noben, Martine Maes, Rob Lavigne

**Affiliations:** 1 Division of Gene Technology, Katholieke Universiteit Leuven, Heverlee, Belgium; 2 Unit Plant – Crop Protection, Institute for Agricultural and Fisheries Research, Merelbeke, Belgium; 3 Division of Crop Biotechnics, Katholieke Universiteit Leuven, Heverlee, Belgium; 4 Laboratory of Foodborne Zoonoses, Public Health Agency of Canada, Guelph, Ontario, Canada; 5 Department of Molecular and Cellular Biology, University of Guelph, Guelph, Ontario, Canada; 6 Biomedical Research Institute and Transnational University Limburg, School of Life Sciences, Hasselt University, Diepenbeek, Belgium; University of Wisconsin, Food Research Institute, United States of America

## Abstract

The bacterium ‘Dickeya solani’, an aggressive biovar 3 variant of *Dickeya dianthicola*, causes rotting and blackleg in potato. To control this pathogen using bacteriophage therapy, we isolated and characterized two closely related and specific bacteriophages, vB_DsoM_LIMEstone1 and vB_DsoM_LIMEstone2. The LIMEstone phages have a T4-related genome organization and share DNA similarity with *Salmonella* phage ViI. Microbiological and molecular characterization of the phages deemed them suitable and promising for use in phage therapy. The phages reduced disease incidence and severity on potato tubers in laboratory assays. In addition, in a field trial of potato tubers, when infected with ‘Dickeya solani’, the experimental phage treatment resulted in a higher yield. These results form the basis for the development of a bacteriophage-based biocontrol of potato plants and tubers as an alternative for the use of antibiotics.

## Introduction

The plant pathogenic *Dickeya* spp. (formerly known as *Erwinia chrysanthemi* or *Pectobacterium chrysanthemi*, [1]) are Gram-negative, non-sporulating, facultative anaerobic bacteria of the family *Enterobacteriaceae*, which characteristically produce pectinolytic enzymes during infection. Along with other pectinolytic bacteria such as *Pectobacterium atrosepticum* and *Pectobacterium carotovorum* subsp. *carotovorum*, they are a major cause of potato tuber soft rot during storage and blackleg disease in the field [2]–[4]. Samson and colleagues (2005) differentiated six species within the genus *Dickeya*, namely *D. zeae*, *D. dadantii*, *D. chrysanthemi*, *D. dieffenbachiae*, *D. dianthicola* and *D. paradisiaca* [1]. Of these six, only *D. paradisiaca* has not been isolated from potato [3], and *D. dianthicola* has been the main species found in Europe. Recently a new, more virulent *Dickeya* type, belonging to biovar 3 of *E. chrysanthemi*, was described and is tentatively named ‘Dickeya solani’ [3], [5]. This *Dickeya* type has become the predominant cause of blackleg of potato in certain European countries [3], [5], [6]. At this moment no chemical disease control measures are available for *Dickeya* and infected batches of potatoes are declassified or discarded, resulting in significant economic losses [3].

Traditionally, a first diagnostic tool for the identification of *Pectobacterium* and *Dickeya* is a PCR analysis based on the *pelY* and the *pelADE* gene cluster, respectively [7], [8]. For *Dickeya* spp., sequence data of both the *recA* gene and *dnaX* were used for phylogenetic analysis of the different species in this genus and these data support the designation of the new species ‘Dickeya solani’ [5], [9]. Recently, a new molecular tool was developed for the identification of this species specifically, a real-time PCR of the virulence gene *fliC* (Van Vaerenbergh et al., submitted manuscript).

(Bacterio)phages have been proposed as biocontrol agents for bacterial diseases in plants [10], [11]. However, phage therapy has to overcome several challenges before it can be efficiently used in agriculture (summarized in [12]). In light of these challenges, Balogh and colleagues [11] argue for the application of bacteriophages in controlled and closed environments with a short window of plant susceptibility, where phages can easily access a homogenous target bacterium population and exposure to harsh environments is limited. In addition, both phage and bacterium need to be extensively characterized and efficiently purified.

In the past, phage therapy research has been carried out on various crops, infected with a broad range of bacteria [11], but no research has been published on the control of *Dickeya* spp. with bacteriophages, although the possibility of phage therapy has been suggested by Czajkowski and colleagues [4]. For this bacterial genus, only temperate phages have been described to date, of which only øEC2 has been characterized as a generalized transducing phage with *Dickeya dadantii* 3937j as host [13], [14]. The related bacterium *Pectobacterium carotovorum* has also been investigated in phage therapy trials on calla lily tubers in the greenhouse [15]. On potato, one case of phage therapy has been reported; the application of bacteriophage øAS1 on seed potatoes infected with *Streptomyces scabies* which causes scab [16]. Infected seed tubers were treated with phage and produced progeny tubers with significantly reduced surface scab lesions.

Of all the phage genome sequences present in the NCBI database, less than 5% are of phages infecting plant pathogenic bacteria. For *Dickeya* spp. no phage genomes are available, only for the related genera *Erwinia* and *Pectobacterium* phage genomes are sequenced [17]–[19], illustrating the need for more genome sequence information. In this paper, we report the succesful application of a new phage species, *Dickeya* phage LIMEstone in an agricultural setting, with both *in vitro* and *in vivo* screens. Of this species, two phages were found, LIMEstone1 and LIMEstone2, which infect ‘Dickeya solani’. The microbiological characterization, as well as sequence analysis, deemed the phage isolates suitable for use in phage therapy.

## Results

### Phages LIMEstone1 and LIMEstone2

#### Isolation of bacteria and phage

Bacteria of the genus *Dickeya* were isolated from diseased potato plants and tubers at the diagnostic clinic of the Institute for Agricultural and Fisheries Research (ILVO, Merelbeke, Belgium) as described by Van Vaerenbergh et al. (submitted manuscript). The isolates (Table S1) were characterized based on barcoding of the *fli*C amplicon and TaqMan qPCR specific for ‘Dickeya solani’ (Van Vaerenberg et al, submitted manuscript). Of the 17 *Dickeya* isolates collected in 2008, 16 were identified as the new ‘Dickeya solani’ type and one was designated as *Dickeya dianthicola*.

**Figure 1:**
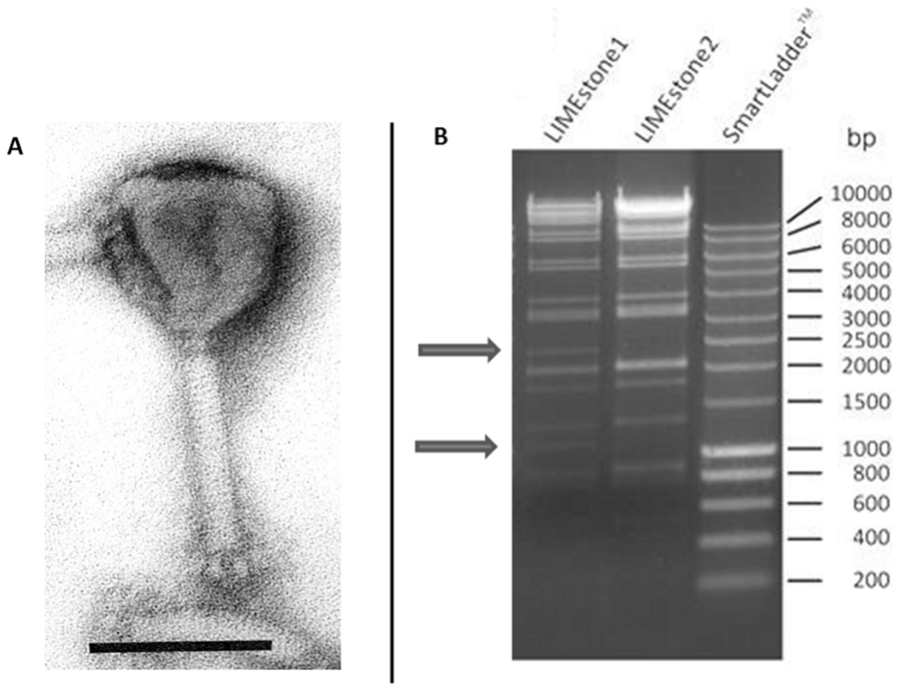
LIMEstone isolates. A) EM picture of phage LIMEstone1. Phage negatively stained with 2% phosphotungstate. Scale bar represents 100 nm. B) HindII restriction digestion of 0.5 and 1.0 µg DNA of LIMEstone1 and LIMEstone2, respectively.

Bacteriophage isolates were made from soil samples from a potato trial field at ILVO after the harvest in September–October 2008. Out of 26 trial plots sampled, 18 contained plants infected with *Dickeya* spp. or *Pectobacterium* spp. Filtrates of the soil were tested for their capacity to lyse a range of *Dickeya* bacteria. In samples of 14 fields, of which 11 were infected and three were uninfected with *Dickeya* spp., phages were found. All phage isolates produced small clear plaques of 1 mm in diameter on ‘Dickeya solani’ strains and restriction digestion of the DNA of the isolates with HindII (Figure 1B) showed two closely related patterns, differing in two bands. These phages were named LIMEstone1 and LIMEstone2 (Leuven ILVO Merelbeke) belonging to the species LIMEstone (scientific names vB_DsoM_LIMEstone1 and vB_Dso_LIMEstone2 as proposed by [20]). Phage isolates belonging to the LIMEstone species were also predominant in soil samples collected from the same fields in 2009 and 2010 (data not shown), isolated according to the same protocol as in 2008. Based on the restriction patterns of the isolates, which were very similar to that of LIMEstone1 and LIMEstone2, it was decided not to further investigate these phages.

#### General characteristics of LIMEstone1 and LIMEstone2

LIMEstone was found to be a member of the *Myoviridae* by transmission electron microscopy (Figure 1A). With an icosahedrical head of 91.4 nm and tail dimensions of 113.8×17 nm, its morphology is similar to that of *Salmonella* phage ViI [21]. A collar was visible (20×2 nm) and several short tail spikes of 12 nm in length. The head volume is smaller than the prolate head of phage T4 (119.5×86 nm), which suggests a smaller genome size for LIMEstone1.

Adsorption and one-step-growth assays were performed for LIMEstone1 and LIMEstone2 isolates to assess the infection parameters (Figure S1). For both phages, more than 99.9999% of phages were irreversibly adsorbed to the host cell within one minute. Upon comparison of the adsorption constant k [k = (2.3/(B*t))*log(P_0_/P), with B the bacterial titer at time zero and t the time], LIMEstone2 (k at 1 min = 2.05×10^−8^ ml/min) appears to adsorb marginally faster than LIMEstone1 (k at 1 min = 9.53×10^−9^ ml/min) and more rapid than reported for T4 (2.4×10^−9^ ml/min) [22]. In the one-step-growth assay, the latent period of LIMEstone1 was determined at 60 minutes with a burst size of 160. The latent period of LIMEstone2 was 65 minutes and about 100 new particles were released. These variations are minor once more indicating the relatedness of LIMEstone1 and LIMEstone2, belonging to one proposed species, LIMEstone.

The viability of LIMEstone in a range of environmental conditions was assessed. Phage were stable at temperatures from 4°C to 37°C in phage buffer, but the titer decreased by three log_10_ units upon storage at 50°C for 24 hours. All viable phage were lost after freezing of the sample. LIMEstone was also stable from pH 4 to 11 for 24 hours.

#### Host range analysis

A collection of *Dickeya* strains (Table S1) was assembled to test the host range of phages LIMEstone1 and LIMEstone2. From the reference set of Van Vaerenbergh et al (submitted manuscript PONE-D-11-23125), two strains per *Dickeya* species were chosen, the type strain and a strain isolated from potato, except for *D. paradisiaca* which has not been found on potato. This collection was supplemented with the 17 strains discussed earlier in this study and with older isolates from the culture collections of the plant clinic of ILVO (GBBC numbers) and of the diagnostic clinic in The Netherlands (PD and PRI numbers).

Only the ‘Dickeya solani’ type was found to be susceptible for infection with both LIMEstone1 and LIMEstone2, with 100% of the strains showing lysis and plaques (Table S1). The isolates of *Dickeya dianthicola* from Belgium and The Netherlands were not infected by either phage, but showed a clear lysis zone when a phage suspension of 10^9^ pfu or higher was spotted on a bacterial lawn. There was no lysis observed when a dilution was spotted and no plaques were formed. This is probably ‘lysis from without’ and not true infection. Of the global *Dickeya* collection, *D. dianthicola*, *D. dadantii*, *D. dieffenbachiae* and *D. chrysanthemi*, all showed this lysis from without, but no phage amplification. The last *Dickeya* species, *D. zeae*, was not infected with either phage and showed no lysis from without, as did a number of environmental isolates identified as *Pectobacterium* spp.

### The genome and proteome of LIMEstone1

#### Genome organization

**Figure 2:**
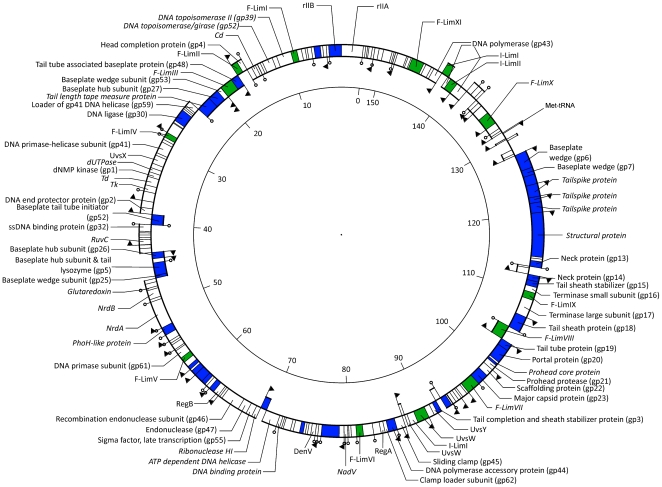
The genome of phage LIMEstone1 (152,427 bp). The inner ring represents ORFs on the forward strand, the outer ring the reverse strand. Proteins in italics show no sequence similarity with T4. ORFs in blue are confirmed as structural proteins, putative homing endonucleases are depicted in green. Promoters are indicated with arrows, factor-independent terminators with stem-loop structures.

Genome sequencing of bacteriophage LIMEstone1 (GenBank accession number HE600015) revealed a genome of 152,427 bp and a G+C content of 49.2%, probably circularly permuted (Figure 2). A total of 201 open reading frames (ORFs) were predicted on both strands and one tRNA (Met-tRNA, anticodon CAT). Of these ORFs, 64 could be linked to bacteriophage T4 based on BLASTP similarity. To another 29 ORFs a putative function could be assigned, leaving 54% of unknown ORFs. A distribution of functional regions, typical for T4-related phages, is observed in phage LIMEstone1, where no well-defined early, middle or late region of transcription was found.

Interestingly, DNA homology has been observed between LIMEstone1 and *Shigella* phage phiSboM-AG3 [23], *Salmonella* phage ViI [21] and *E. coli* phage CBA120 [24]. The genome of LIMEstone1 showed a DNA homology of 69.1% with phiSboM-AG3 sharing 174 genes out of 201. Similarity with ViI and CBA120 is less with 58.7% and 59.4% respectively. The gene order is strongly conserved between the four phages, with a few insertions present, due to the other's larger genome sizes (ViI 157,061 bp, CBA120 157,304 bp and phiSboM-AG3 158,006 bp). The overall similarity between these phages is quite remarkable, considering all four of them infect different bacterial genera.

#### Regulatory elements

In phage T4, transcription is mediated by three classes of promoters – early, middle and late [25]. These classes were also identified in LIMEstone1 based on sequence similarity of the −35 and −10 boxes with those of T4. Five putative early promoters, five middle and 33 late promoters were located in intergenic regions on both strands (indicated in Figure 2 with arrows). Another four putative promoters were found based on the −10 box of *Dickeya dadantii* 3937 in which only five promoters have been annotated [26]. A total of 31 rho factor-independent terminators were identified throughout the genome of LIMEstone1, located on both strands (Figure 2).

#### Mobile elements

Homing endonucleases are mobile genetic elements, which recognize a DNA target site and generate single or double-stranded breaks in the genome to insert themselves in the target genome [27]. While their exact function is not known, Goodrich-Blair and Shub suggest they confer a selective advantage to the flanking sequences in the phage genome [28]. In phage LIMEstone1, 14 homing endonucleases were found, representing 10% of the genome (Table 1). This is comparable to the 15 homing endonucleases found in phage T4 (reviewed in [29] and [30]), but considered an oddity among the other T4-related phages. Three putative introns were identified in LIMEstone1, designated I-LimI, I-LimII and I-LimIII [31], the first two in the DNA polymerase gene, the third in *uvs*W. These two genes are functionally essential and strongly conserved between the T4-related phages and make thus an good target for intron homing [30]. The 11 free-standing homing endonucleases found in LIMEstone1 could be divided into three groups, the endonucleases encoding a GIY-YIG motif, the HNH-containing endonucleases and the Hef-like endonucleases (homing endonuclease-like function) [32], [33] and were named F-LimI through F-LimXI according to Roberts and colleagues [31].

**Table 1:** Mobile elements in the genome of LIMEstone1.

ORF	HEase name	Phage homolog (phage name)	Intron or free-standing	Group	Target gene or downstream gene^a^
ORF17	F-LimI	SegB (T4)	Free-standing	GIY-YIG	ORF18
ORF12	F-LimI	MobB/C/D/E	Free-standing	GIY-YIG	ORF13 (DNA topoisomerase II)
ORF22	F-LimII	SegD (133)	Free-standing	GIY-YIG	ORF21 (Head completion protein)
ORF24	F-LimIII	Hef (Acj9)	Free-standing	Hef-like	ORF25 (Baseplate wedge subunit)
ORF36	F-LimIV	MobC (phiSboM-AG3)	Free-standing	HNH	ORF33
ORF76	F-LimV	MobE (phiSboM-AG3)	Free-standing	HNH	ORF75 (DNA primase)
ORF114	F-LimVI	I-TevI	Free-standing	GIY-YIG	ORF113
ORF123	I-LimI	MobB/D	Intron	GIY-YIG	ORF122-124 (UvsW)
ORF137	F-LimVII	Hef (Acj9)	Free-standing	Hef-like	ORF136
ORF145	F-LimVIII	Hef (CP220)	Free-standing	Hef-like	ORF144 (Tail tube protein)
ORF148	F-LimIX	MobE (T4)	Free-standing	GIY-YIG	ORF147 (Terminase large subunit)
ORF171	F-LimX	Hef (Acj9)	Free-standing	Hef-like	ORF170
ORF179	I-LimII	MobE (Acj9)	Intron	GIY-YIG	ORF178-180-182 (DNA polymerase)
ORF181	I-LimIII	MobE (phiAS5)	Intron	HNH	ORF178-180-182 (DNA polymerase)
ORF186	F-LimXI	SegB (T4)	Free-standing	GIY-YIG	ORF187

a target gene for intron encoded homing endonucleases, downstream gene for free-standing endonucleases.

#### Structural proteome

The virion particle of LIMEstone1 consisted of at least 39 proteins, as verified by mass spectrometry (Table 2). Of these proteins, 27 had a function assigned based on sequence similarity with other phage proteins, in addition to 12 unknown structural proteins. As expected, the most abundant proteins in the sample were the major capsid protein gp23 (ORF138) and the tail sheath protein gp18 (ORF146). There is one structural region found in the genome, from ORF138 on the complementary strand (major capsid protein gp23) to ORF163 (baseplate wedge subunit gp6). In this region, the gene order is largely conserved between LIMEstone1 and T4. Four structural proteins of T4 Gp8, Gp10, Gp11 and Gp12, could not be found in phage LIMEstone1, but structural proteins were present in the corresponding locations to substitute the function of the missing T4 proteins. Two mobile elements were also located in this region; F-Lim-VIII located on the opposite strand between the tail tube and portal proteins (ORF144 and ORF146) and F-LimIX between the two subunits of the terminase complex (ORF147 and ORF149). There is another insertion of two hypothetical proteins with their own promoter and terminator between the two neck proteins, ORF151 and ORF154.

**Table 2:** Structural proteins of LIMEstone1 as confirmed by mass spectrometry.

ORF	Putative protein	Size of protein (kDa)	Protein coverage^a^	N° of unique peptides recovered
2	rIIB (T4 rIIB)	57.38	3.85%	1
6	Head outer capsid protein (T4 Hoc)	27.46	21.93%	4
23	Tail tuber associated baseplate protein (T4 gp48)	36.12	20.19%	4
26	Baseplate hub subunit (T4 gp27)	52.61	19.05%	6
27	Tail length tape measure protein	70.95	15.72%	7
32	DNA ligase (T4 gp30)	53.26	2.95%	1
50	Baseplate tail tube initiator (T4 gp54)	35.06	24.52%	7
59	Baseplate hub subunit (T4 gp26)	30.56	6.34%	1
61	Baseplate hub subunit & tail lysozyme (T4 gp5)	58.18	7.46%	2
62	Baseplate wedge subunit (T4 gp25)	14.04	19.05%	2
69	PhoH	31.47	3.93%	1
78	Unknown structural protein	13.15	28.21%	3
80	Unknown structural protein	20.28	12.17%	2
81	Unknown structural protein	40.85	55.20%	23
82	Unknown structural protein	18.76	19.63%	4
85	Unknown structural protein	22.75	16.98%	4
93	Unknown structural protein	28.32	40.78%	8
102	Unknown structural protein	17.31	35.57%	5
108	vWa containing protein	81.06	11.44%	6
119	DNA polymerase accessory protein (T4 gp44)	37.24	4.26%	1
127	Tail completion & sheath stabilizer protein (T4 gp3)	18.52	6.63%	1
129	Unknown structural protein	25.03	18.18%	2
138	Major capsid protein (T4 gp23)	48.02	73.41%	18
141	Prohead core protein	38.57	8.91%	1
143	Portal protein (T4 gp20)	63.29	38.19%	17
144	Tail tube protein (T4 gp19)	19.99	27.68%	4
146	Tail sheath protein (T4 gp18)	68.80	49.53%	27
151	Neck protein (T4 gp14)	24.97	35.19%	6
154	Neck protein (T4 gp13)	28.72	17.20%	4
157	Structural protein	177.55	24.38%	21
158	Tailspike protein	54.76	22.45%	17
159	Tailspike protein	21.59	11.76%	2
160	Tailspike protein	53.69	25.00%	8
161	Fibritin (T4 Wac)	42.69	50.62%	11
162	Baseplate wedge subunit (T4 gp7)	33.36	4.93%	1
163	Baseplate wedge subunit (T4 gp6)	64.75	18.07%	7
169	Unknown structural protein	18.45	24.42%	3
173	Unknown structural protein	17.33	11.84%	1
174	Unknown structural protein	16.62	12.50%	1

a Coverage of the protein sequence by the peptides recovered during ESI-MS/MS.

The other structural proteins were scattered throughout the entire genome on both strands, with some components of baseplate and tail tube grouping together (ORF23-27; ORF59-62). Between ORF78 and ORF85, a small group of structural proteins were clustered together, but no specific functional predictions could be made.

Despite the high number of structural proteins recovered, two proteins of the virion (the head completion protein gp4 and the baseplate wedge subunit gp53) were not found by mass spectrometry. Also, the analysis showed four unexpected proteins with a low, yet significant peptide coverage (<5%), rIIB (ORF2), the DNA ligase gp30 (ORF32), PhoH (ORF69) and the DNA polymerase accessory protein gp44 (ORF119), which might suggest co-infection of these four proteins with the DNA as it is injected.

### Phage therapy biocontrol on potato

#### Virulence test on seed tubers

This test was designed to investigate whether the anti-bacterial effect of phages LIMEstone1 and LIMEstone2 on ‘Dickeya solani’ is also present *in vivo*, i.e. on tubers, and to quantify this effect.

In a preliminary experiment, infection conditions for the pathogen, ‘Dickeya solani’ strain LMG 25865, were determined. A concentration of 10^5^ colony forming units (cfu) infiltrated per tuber combined with incubation at 28°C in a micro-aerophilic environment were determined as ideal positive control conditions, since this ensured visible infection of the tubers in more than 90% of the cases.

**Figure 3:**
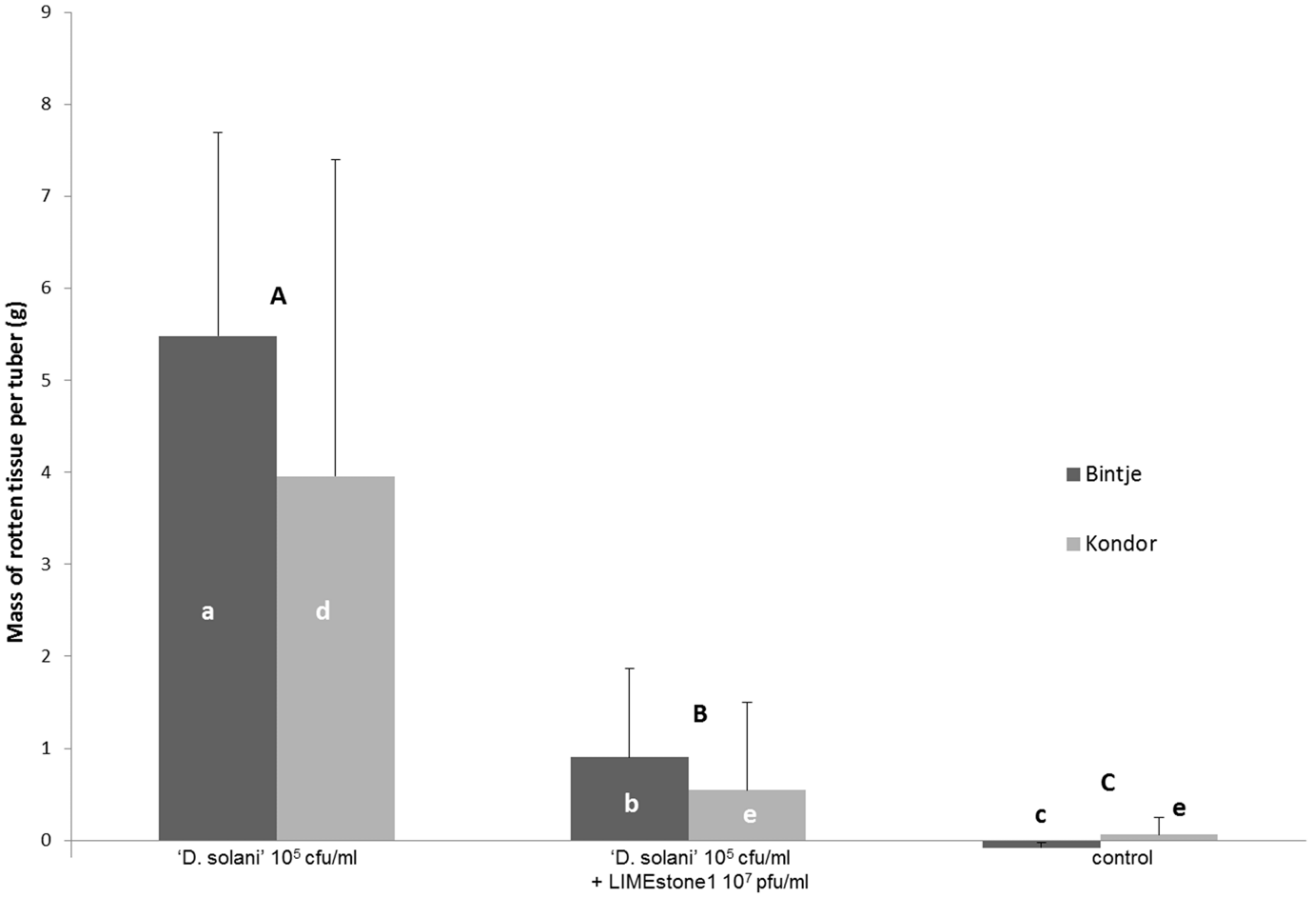
Phage therapy assay on potato tubers cv. Bintje and Kondor. Tubers treated with ‘Dickeya solani’ strain LMG 25865 were compared with phage treated tubers and with a water-treated control. Error bars indicate standard deviation. Significant differences were tested with the Kruskal-Wallis multiple comparison tests at p<0.05 en the Mann-Whitney U test for comparison of two samples. Letters indicate significant differences, capitals between treatments, small letters within cultivars.

The effect of treatment with phages on the rotting of potato tubers (cultivar (cv.) Bintje) was assessed under these micro-aerophilic conditions (Figure 3). This cultivar was chosen because it is the predominant cultivar in Belgium, with 42% of the total acreage in 2010 (National Institute for Statistics Belgium data). Phages LIMEstone1 and LIMEstone2 were added at a multiplicity of infection (MOI) of 100 each to 20 tubers inoculated with LMG 25865. Looking at the number of rotten tubers for the positive control, 18 out of 20 tubers displayed rot. For the phage treated tubers the incidence of infection decreased significantly to 12 out of 20 for LIMEstone1 and 8 out of 20 for LIMEstone2. Moreover, a significant decrease in disease severity per tuber was observed after phage treatment. Both with LIMEstone1 and LIMEstone2, less than 10% rotten tissue per tuber was found (less than 1 g per tuber), calculated on the weight of the tuber before treatment and after the rotten tissue was scraped off, while the positive control group, which was only infected with bacteria, had an average of over 40% (5.5 g) rot per tuber (p values of 0.005073 and 0.000968, respectively). Between the two phages, no significant difference was observed in the amount of rotten tissue (p = 1.0). It can be concluded that the application of a surplus of bacteriophages can significantly reduce both the number of rotten tubers and the extent of tuber rotting caused by *D. solani* strain LMG 25865. For LIMEstone1, this test was repeated on a different cultivar of potato, Kondor. At an MOI of 100 rotting of the potato tubers was significantly reduced from over 20% (4 g/tuber) to less than 5% (0.5 g) rot (p = 0.041242) (Figure 3). An MOI of 10 was also tested (data not shown) and also showed a decrease in the amount of rotten tissue, but this was not statistically significant, neither between the positive control and an MOI of 10 (p = 0.256840), nor between an MOI of 10 and an MOI of 100 (p = 0.794024). The number of rotten Kondor tubers also showed a decrease after phage treatment with an MOI of 100.

Comparing the data of Bintje and Kondor (Figure 3) there was a variation in the percentage of tissue rot per tuber (averages of 23.5% and 42.5% respectively). This was due to the difference in size of the tubers between these two cultivars, because there was no significant difference between the two cultivars (p = 0.815129).

#### Field trial

The effect of phage treatment on potato tuber and plant growth was examined in a field trial. A latent infection of seed tubers with ‘D. solani’ was mimicked by vacuum infiltration of the tubers with a bacterial suspension. Next, a suspension of LIMEstone1 was nebulized over a batch of the infected tubers, to simulate a conveyor belt in a farm environment and the phage treated tubers were air dried. Three treatments were compared: an untreated control (treatment A), a positive control with only bacteria (treatment B), and co-treatment of bacteria and phage (treatment C). Tubers were kept out of direct sunlight until the moment of planting to avoid the interference of UV light in the experiment.

The emergence of the plants and disease incidence was monitored throughout the growing season. The first signs of infection, darkening and wilting of the shoot tips and young leaves, were observed 42 days after planting, for two plants of treatment B and one plant in treatment C. In the course of the next 20 days, more than 90% of the plants of treatment B showed symptoms of *Dickeya* infection, ranging from wilting, to leaf necrosis and stem rot (blackleg). In treatment regime C, disease incidence was a little less with 85% of plants displaying symptoms. A greater difference in disease severity was observed between treatment B and C, with none of the diseased plants of treatment C presenting stem rot, only wilting and leaf necrosis. For the control plants, no symptoms were observed throughout the growing season.

**Figure 4:**
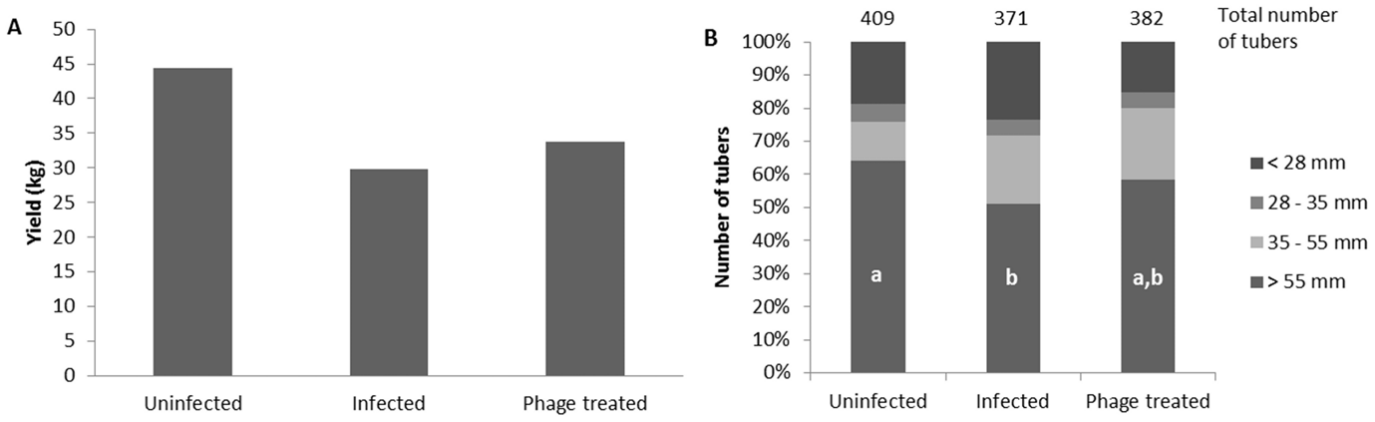
Field trial results. A) Total yield of the tubers in mass. B) Tuber size distribution in percentages of the total number of tubers. In the bars of fraction >55 mm, letters indicate statistical significance (p<0.05) as determined with the Kruskal-Wallis non-parametric test. Other fractions are not significantly different from each other.

Tubers were harvested from the field 82 days after planting. The total yield for each treatment was 44.4 kg for the untreated plants (A), 29.9 kg for the ‘Dickeya solani’-treated plants (B), and 33.8 kg for the plants treated with phage LIMEstone1 (C) (Figure 4A). With a difference of 3.9 kg, phage treatment of infected potato tubers led to a 13% yield increase. This increase was mostly due to the size distribution of the tubers. The total number of tubers harvested from treatment C (382) was only 3% higher than the number of tubers from treatment B (371), both significantly less than the 409 tubers collected from group A.

The harvested tubers were divided into four groups according to their sizes, smaller than 28 mm, between 28 and 35 mm (seed tuber size), between 35 and 55 mm, and bigger than 55 mm (fry cut) (Figure 4B). This last category had the largest difference between treatments. As expected, the untreated plants had the highest number of tubers in this size range (262). For the plants inoculated with bacteria, this was significantly less, with 190 tubers (p = 0.044533). The number of tubers of the bacteria/phage treated plants was intermediate (223) and was not significantly different from either the control or the bacteria treated plants (p values of 0.820896 and 1.0, respectively) (Figure 4B).

For the control A, one rotten tuber was found, but this was not caused by ‘D. solani’, as confirmed by *pelADE* PCR and *fliC* qPCR on the rotted tissue. For treatments B and C, 9 and 6 ‘D. solani’ rotted tubers were collected respectively, a significant difference from the control treatment A. The difference between the number of rotten tubers of B and C, on the other hand was not big enough to be significant.

## Discussion

*Dickeya* spp. are of increasing concern in potato production in various parts of Europe [3]. It was apparent during our bacterial isolation tests that ‘D. solani’ has replaced *D. dianthicola* as the most prevalent pathotype. In 2008, less than 10% of the isolated *Dickeya* strains belonged to *D. dianthicola* (only 1 out of 17 strains described in this paper). In this respect, it is logical that the phages LIMEstone1 and LIMEstone2, isolated in the same year, specifically infect ‘D. solani’ and that no *D. dianthicola* phages have been isolated. These two were the only *Dickeya* phages that were isolated during the course of this study. Since they were isolated in three consecutive years (data not shown), it can be said that they are stable in this environment. They also infect 100% of the ‘Dickeya solani’ strains, which offers an explanation for the low diversity in phage types, as they might out-compete other phages. The broad host range of LIMEstone within ‘D. solani’ and its suspected abundance in the environment are characteristics of the group of T4-related phages. These are widespread around the globe and infect an array of different hosts, ranging from enterobacteria found in sewage to cyanobacteria from marine environments. Another reason for the low diversity of phages found could be the culturing method used for isolation, which seems to favor members of the *Caudovirales* phage family.

The genome of phage LIMEstone1 was sequenced revealing a T4-related gene organization (*Tevenvirinae*), belonging to the proposed new genus of the ‘ViI-like viruses’ [23] which includes the type phage ViI, phiSboM-AG3 and CBA120. Gene order of these phages is strongly conserved in LIMEstone1. A specific feature for LIMEstone1 is the presence of a large number of homing endonucleases (not unusual for T4-related phages). As of yet, no explanation can be offered for this.

The 39 structural proteins recovered for LIMEstone1, is similar to the 41 structural proteins found for ViI [21], and all the 12 structural proteins of unknown function of LIMEstone1 have a structural counterpart in ViI. Like ViI, LIMEstone1 encodes three potential tailspike proteins. Two of them (ORF158 and 159) show significant similarity to the conserved N-terminal regions of the ViI tailspike proteins ViI_170c and ViI_171c respectively. The third one (ORF160) shows great similarity to the N-terminal domain of the putative tail fiber of ViI (ViI_173c), and the N-terminal domain of a tailspike protein of CBA120 (ORF213). Since there are no tail fibers visible on the electron micrograph and no long tail fiber genes are found, we assume ORF157 is indeed a tailspike protein. The acetyl esterase containing tailspike protein of ViI (ViI_172c) was not found in LIMEstone1. Since these acetyl esterases are thought to specifically target the Vi antigen in the capsule of *Salmonella* (not present in *Dickeya* spp.), the absence of this tailspike in the *Dickeya* phage LIMEstone1 may be explained.

The extensive characterization of phages LIMEstone1 and LIMEstone2 revealed their suitability for phage therapy. They both infect all of the ‘D. solani’ strains, showed rapid adsorption and a large burst size. In addition, bio-informatic analysis of the LIMEstone1 genome showed no known toxic genes, potential allergens or integrases. Since the genome of LIMEstone2 is very similar to LIMEstone1 as determined by restriction digestion analysis, this was also assumed for LIMEstone2. No host DNA was found during sequence analysis and no host proteins during mass spectrometry, therefore no generalized transduction assays were performed. Also, T4 type phages are considered safe for administration to humans and animals because they do not cause adverse effects and are not prone to lysogenic conversion and transduction [34].

In a ‘proof-of-concept’ experiment, the effect of phage on the rotting of potato tubers was studied under conditions most favorable for disease development. The addition of an 100-fold surplus of phage compared with the bacterial inoculum in a tuber model of disease significantly decreased both the number of rotten tubers and the amount of rotted tissue in the diseased tubers. The results obtained were the same for phages LIMEstone1 and LIMEstone2, and for the different potato cultivars used (Kondor and Bintje). A decrease in the number of phage added, resulted in less suppression of rotting. This suggests that phage therapy can only work when a sufficiently large number of phage are added. Taking this into account, as well as the low bacterial titer that can lead to disease development and a phage titer that is economically feasible to produce, we chose to spray the tubers with 10^7^ pfu/ml of LIMEstone1 in the field trial.

The results of the field trial gave a first indication that phage therapy before planting of the seed tubers provides protection against a symptomless bacterial infection. The increase in yield with phage treatment was 13% when all tubers were inoculated with ‘D. solani’. Also, some of the rotten tubers found were not infected with *Dickeya*, but with *Pectobacterium*. Isolating phages against other soft rot bacteria such as *P. atrosepticum* and *P. carotovorum* subsp. *carotovorum* will undoubtedly increase the success of a therapy by using a cocktail of different phages.

The timing of phage application is also essential for a good result. One batch of tubers was sprayed with a phage suspension only minutes before planting, making sure the tubers went into the ground while still wet (data not shown). Disease development in the field was more severe for these plants and the yield was considerably less than without this phage treatment. This was probably due to the water film on the tubers creating a micro-aerobic environment, which lowers plant defenses and promotes the infection process of *Dickeya* spp. It is thus important to dry the phage-treated tubers well before planting.

In conclusion, we can say that phages LIMEstone1 and LIMEstone2 belong to a group of globally abundant T4-related phages and have all the characteristics of a successful therapeutic agent in an agricultural setting. The phage therapy experiments on potato in the lab and in the field, support this statement and can be important for policymakers in the European Union (and elsewhere) to accept phage therapy as a means of biocontrol on crops.

## Materials and Methods

### Bacteria and growth media

Bacterial isolates were provided by the diagnostic unit of ILVO and typed as previously described by Van Vaerenbergh et al. (submitted manuscript PONE-D-11-23125). Strains were confirmed as *Dickeya* spp. by *pel*ADE PCR or as *Pectobacterium* spp. by *pel*Y PCR with primers as previously described [7], [8]. Further typing of the *Dickeya* strains was done by barcoding of the *fli*C gene and a TaqMan qPCR of the same gene for detection of the ‘Dickeya solani’ type (Van Vaerenbergh et al., submitted manuscript PONE-D-11-23125). Strains were grown in liquid culture in LB medium at 28–30°C or on plates of LB with 1.5% agar; LB with 0.7% agar was used for the overlays.

### Bacteriophage isolation, amplification and purification

Bacteriophages LIMEstone1 and LIMEstone2 were isolated from 20 g soil samples, taken from the same potato field from which some of the bacterial strains were isolated. The soil was shaken for 30 min in sterile, demineralized water and filtered over a 0.45 µm membrane (Millipore). Next, the filtrate was centrifuged for 90 min at 28,000× g (Sigma 3K30, fixed angle rotor 12156-H, B. Braun Biotech, USA) and the pellet was resuspended in phage buffer (10 mM Tris-HCl pH 7.5; 10 mM MgSO_4_; 150 mM NaCl). This suspension was spotted on a plate with a soft agar overlay of a ‘Dickeya solani’ culture. The resulting lysis zones were picked up with sterile toothpicks and three successive single plaque isolations were performed using the standard agar overlay method [35]. Phages were amplified in liquid LB medium; ‘D. solani’ strain GBBC 2072, randomly selected from the collection, was grown to an optical density at 600 nm (OD_600_) of 0.6 and phages were added. The culture was left to lyse overnight. Any remaining cells were lysed with chloroform (0.5% final concentration) and kept at room temperature for at least two hours. Cell debris was removed by centrifugation for 30 min at 8000× *g* in a Sorvall Legend RT+ centrifuge with swing-out 4-place rotor, type 75006445 (Thermo Scientific, Waltham, MA, USA). The supernatant was filtered in a filter funnel (Nalgene) with a cellulose nitrate membrane of a pore size of 0.2 µm. Phage purification was carried out with anion exchange chromatography using a CIM® monolithic disc (QA and DEAE) (BIA Separations, Ljubljana, Slovenia) on an AKTA FPLC system (GE Healthcare, Little Chalfont, UK). Data was analyzed with UNICORN™ 5.01 software.

### Electron microscopy

Phage particles were pelleted by centrifugation for 1 h at 25,000× g and washed twice in 0.1 M ammonium acetate (pH 7.0) using a Beckman (Palo Alto, CA, USA) high-speed centrifuge and a JA-18.1 fixed angle rotor. They were then deposited on copper grids with carbon-coated Formvar films, stained with 2% (w/v) potassium phosphotungstate (pH 7.0) and examined in a Philips EM 300 electron microscope [36].

### Host range and general characterization

The host range of phages LIMEstone1 and LIMEstone2 was tested by standard plaque assays and by spotting of a phage suspension on a bacterial lawn. The titer of the suspension ranged from 10^6^ pfu/ml to determine infectivity to 10^10^ pfu/ml to assess lysis from without. The *Dickeya* strains used in the host range assay are summarized in Table S1. In adsorption experiments, the host strain GBBC 2072 was grown to an OD_600_ of 0.4 and infected with phages at a multiplicity of infection (MOI) of 0.001. Immediately after infection, a 100 µl sample was taken and transferred into 850 µl LB medium supplied with 50 µl CHCl_3_. This was repeated every minute. These mixtures were shaken gently for 15 minutes to lyse any remaining bacteria. The supernatant was titrated to determine the amount of non-adsorbed or reversibly adsorbed phages. One-step-growth assays were performed according to [37]. Phage stability was tested by incubating a phage suspension of 10^6^ pfu/ml in phage buffer for at different temperatures or in pH buffer ranging 1 to 13 (150 mM KCl, 10 mM Na_3_citrate, 10 mM H_2_BO_3_ with NaOH or HCl).

### Genome and proteome

#### DNA isolation and sequencing

DNA was isolated according to [38]. The genome was sequenced by the McGill University and Génome Québec Innovation Centre (Montréal, QC, Canada) using (454 technology) to 36-fold coverage. The sequence was reordered so that it was collinear with that of Salmonella phage ViI prior to annotation.

#### ‘In silico’ analysis

The genome of LIMEstone1 was scanned for potential open reading frames (ORFs) with Kodon (Applied Math, Sint-Martens-Latem, Belgium), ORF Finder [39] and GeneMark.hmm software [40]. Shine-Dalgarno sequences were verified manually upstream from each annotated ORF. Functional bioinformatic annotation was carried out by comparing translated ORFs in a BLASTP [41] analysis against the nonredundant GenBank protein database and using the HHPred prediction software [42]. The presence of transmembrane domains was verified with TMHMM software [43], signal peptides were identified with SignalP [44] and coiled coils were found using COILS [45]. Host promoter regions were identified using the Nostradamus prediction program [46], MEME/MAST [47] and PHIRE [48] software and with Fuzznuc [49] based on the promoter consensus sequences of bacteriophage T4. Terminators were identified as palindromic repeat regions with a U-rich stretch and found with TransTerm [50] and Mfold [51]. Nucleotide similarity between phages was compared using the Stretcher algorithm [52].

The annotated genome sequence of LIMEstone1 was deposited in the EMBL GenBank database under the name vB_DsoM_LIMEstone1 with accession number HE600015.

#### Proteome

Structural proteins of LIMEstone1 were identified by SDS-page gel electrophoresis, cutting out slices of the gel, subsequent trypsinization and ESI-MS/MS as previously described in [53].

### Phage therapy on potato

Potato tubers (*Solanum tuberosum*) used for all bio-assays were prebasic or basic seed tubers, that were already tested for the presence of two quarantine bacteria, *Clavibacter michiganensis* subsp. *sepedonicus* and *Ralstonia solanacearum*. These tubers, from the cultivars Bintje and Kondor, were sanitized before testing with *Dickeya solani* by washing them in 0.5% NaOCl household grade, for 10 min and subsequent washing with tap water. They were air dried and stored at 16°C.

#### Virulence test

All tubers were weighed before the experiment. Next, they were incised at the opposite side from the stolon end and a cap was removed. At this spot, 100 µl of bacterial suspension (*Dickeya solani* strain LMG 25865) in sterile demineralized water was pipetted or 100 µl of sterile water for the negative control. The tubers were left to rest until the fluid was absorbed into the tissue, taking about 10 minutes. For the phage therapy assays, 100 µl of phage suspension (LIMEstone1 or LIMEstone2) in phage buffer was added to the cut-out or 100 µl of sterile phage buffer for the positive control. The tubers were again left until all fluid was absorbed and the cap was secured on the tuber with a sterile toothpick. They were placed one by one in plastic containers on a humid paper tissue and incubated at 28°C in a vacuum incubator (Memmert GmbH, Schwabach, Germany) for 70 hours. Rotten tissue was subsequently scraped off the tubers and the weight of the remaining tuber tissue was determined.

#### Field trial

Sanitized potato tubers of the cultivar Kondor were submerged in a cell suspension of *D. solani* strain LMG 25865 (10^8^ cfu/l) in a vacuum incubator (50 mb, 28°C) for 30 min, and were then air dried for 30 min. A suspension of 10^10^ pfu/l of LIMEstone1 was sprayed on the tubers and left to dry for two hours before planting (150 ml for 32 tubers). Tubers were planted on May 11^th^ 2011, in blocks of eight tubers per treatment, spaced at least 80 cm apart to minimize diffusion effects. The blocks were divided over four rows; tubers were spaced 40 cm apart and planted at a depth of 12 cm. Before emergence of the shoots, the field was treated with the herbicide Roundup® (Monsanto Company, St Louis, MO, USA) according to the manufacturer's instructions. During the growing season, weekly applications with the fungicides Tattoo® C (Bayer CropScience, Monheim am Rhein, Germany) and Shirlan (Syngenta, Basel, Switzerland) were performed to prevent the emergence of the potato disease, *Phytophtora infestans*. Tubers were harvested by hand on August 1^st^, rinsed with tap water, weighed and measured.

#### Statistical analyses of data

Figures 3 and 4 were generated with Excel. Statistical analysis were performed with Statistica (Statsoft, Tulsa, OK, USA). Normality of data was assessed with the Shapiro-Wilk and Lilliefors tests at a significance level of 0.05. For the normally distributed data (Field trial weight data), Scheffé's test for multiple comparisons was used. Non-parametric tests were chosen for not normally-distributed data. Comparison of more than two groups was performed using the Kruskal-Wallis non-parametric test. For the data of the virulence test on the cultivar Kondor, the Mann-Whitney U non-parametric test for comparison of two groups was used, because the very low variance of the control group skewed the results of the Kruskal-Wallis test.

## Supporting Information

Figure S1 Adsorption and one-step-growth curves of phages LIMEstone1 and LIMEstone2. A) Adsorption curves of LIMEstone1 and LIMEstone2. P/P0: ratio of free phages to original number of phage added. B) One-step-growth curves of LIMEstone1 and LIMEstone2. Burst sizes are indicated.
(TIF)

Table S1 Bacterial strains and host range of LIMEstone1 and LIMEstone2.
(DOC)
