# Supplementary figures and images for: T4-Related Bacteriophage LIMEstone Isolates for the Control of Soft Rot on Potato Caused by ‘Dickeya solani’
Source: PLoS One. 2012 Mar 7;7(3):e33227. doi: 10.1371/journal.pone.0033227 (PMC3296691; doi:10.1371/journal.pone.0033227)

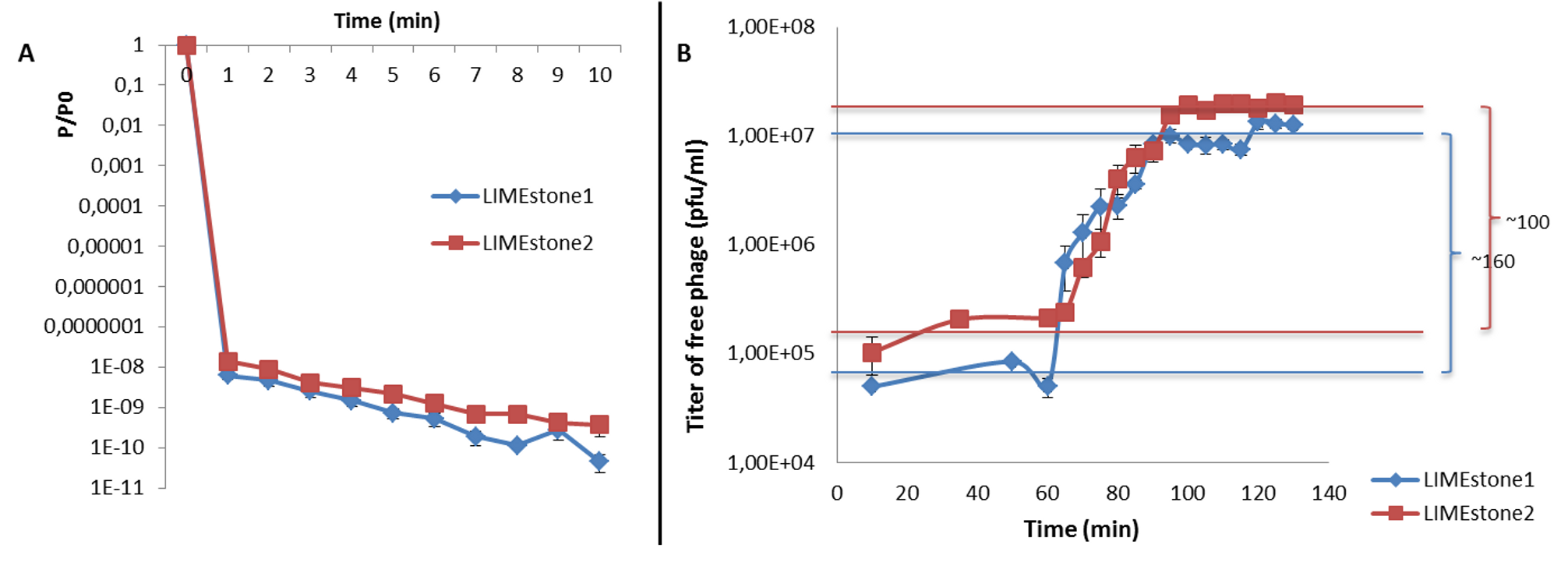

Supplement: Figure S1 — Adsorption and one-step-growth curves of phages LIMEstone1 and LIMEstone2. A) Adsorption curves of LIMEstone1 and LIMEstone2. P/P0: ratio of free phages to original number of phage added. B) One-step-growth curves of LIMEstone1 and LIMEstone2. Burst sizes are indicated. (TIF) [file pone.0033227.s001.tif]
