# Supplementary material for: T4-Related Bacteriophage LIMEstone Isolates for the Control of Soft Rot on Potato Caused by ‘Dickeya solani’
Source: PLoS One. 2012 Mar 7;7(3):e33227. doi: 10.1371/journal.pone.0033227 (PMC3296691; doi:10.1371/journal.pone.0033227)
Supplement: Table S1 — Bacterial strains and host range of LIMEstone1 and LIMEstone2. (DOC) [file pone.0033227.s002.doc]

Table S1: Bacterial strains and host range of LIMEstone1 and LIMEstone2.

| **Strain designation^a^** | **Species** | **Biological origin** | **Geographical origin** | **Year of isolation** | **Genus/species determination** | **LIMEstone1** | **LIMEstone2** |
| --- | --- | --- | --- | --- | --- | --- | --- |
| **Reference set** |  |  |  |  |  |  |  |
| **LMG 2485**, CFBP 1200, NCPPB 453 | *D. dianthicola* | *Dianthus caryophyllus* | UK | 1956 | Van Vaerenbergh, submitted | - | - |
| **PRI 3332** | *D. dadantii* | *Solanum tuberosum* | Peru |  | Van Vaerenbergh, submitted | - | - |
| **NCPPB 3855,** ECH3937 | *D. dadantii* | *Saintpaulia* | France | 1977 | Van Vaerenbergh, submitted | - | - |
| **GBBC 2086**, PRI 1259 | *D. dieffenbachiae* | *Solanum tuberosum* | Germany |  | Van Vaerenbergh, submitted | - | - |
| **NCPPB 3537**, PRI 2125 | *D. dieffenbachiae* | *Dieffenbachia* | USA | 1957 | Van Vaerenbergh, submitted | - | - |
| **LMG 2505**, CFBP 2052, NCPPB 2538, PRI 2131 | *D. zeae* | *Zea mays* | USA | 1970 | Van Vaerenbergh, submitted | - | - |
| **NCPPB 3531**, PRI 3329 | *D. zeae* | *Solanum tuberosum* | Australia |  | Van Vaerenbergh, submitted | - | - |
| **LMG 2804,** CFBP 2048, NCPPB 402, PRI 2118 | *D. chrysanthemi* | *Chrysanthemum* | USA | 1956 | Van Vaerenbergh, submitted | - | - |
| **NCPPB 3533**, PRI 3330 | *D. chrysanthemi* | *Solanum tuberosum* | USA |  | Van Vaerenbergh, submitted | - | - |
| **Potato isolates** |  |  |  |  |  |  |  |
| **LMG 25865**, GBBC 1012 | ‘D. solani’ | *Solanum tuberosum* cv. Première | Belgium | 2007 | *fliC*: ‘solani’; qPCR *fliC*: + | + | + |
| **GBBC 2064** | ‘D. solani’ | *Solanum tuberosum* cv. Bintje | Belgium | 2005 | *fliC*: ‘solani’; qPCR *fliC*: + | + | + |
| **GBBC 2065** | ‘D. solani’ | *Solanum tuberosum* cv. Mona Lisa | Belgium | 2008 | *fliC*: ‘solani’; qPCR *fliC*: + | + | + |
| **GBBC 2066** | ‘D. solani’ | *Solanum tuberosum* cv. Spunta | Belgium | 2008 | *fliC*: ‘solani’; qPCR *fliC*: + | + | + |
| **GBBC 2067** | ‘D. solani’ | *Solanum tuberosum* cv. Bintje | Belgium | 2008 | *fliC*: ‘solani’; qPCR *fliC*: + | + | + |
| **GBBC 2068** | ‘D. solani’ | *Solanum tuberosum* cv. Draga | Belgium | 2008 | *fliC*: ‘solani’; qPCR *fliC*: + | + | + |
| **GBBC 2069** | ‘D. solani’ | *Solanum tuberosum* cv. Eos | Belgium | 2008 | *fliC*: ‘solani’; qPCR *fliC*: + | + | + |
| **GBBC 2070** | ‘D. solani’ | *Solanum tuberosum* cv. Saturna | Belgium | 2008 | *fliC*: ‘solani’; qPCR *fliC*: + | + | + |
| **GBBC 2071** | ‘D. solani’ | *Solanum tuberosum* cv. Dorado | Belgium | 2008 | *fliC*: ‘solani’; qPCR *fliC*: + | + | + |
| **GBBC 2072** | ‘D. solani’ | *Solanum tuberosum* cv. Bintje | Belgium | 2008 | *fliC*: ‘solani’; qPCR *fliC*: + | + | + |
| **GBBC 2073** | ‘D. solani’ | *Solanum tuberosum* cv. Bintje | Belgium | 2008 | *fliC*: ‘solani’; qPCR *fliC*: + | + | + |
| **GBBC 2074** | ‘D. solani’ | *Solanum tuberosum* cv. Désiree | Belgium | 2008 | *fliC*: ‘solani’; qPCR *fliC*: + | + | + |
| **GBBC 2075** | ‘D. solani’ | *Solanum tuberosum* cv. Jaerla | Belgium | 2008 | *fliC*: ‘solani’; qPCR *fliC*: + | + | + |
| **GBBC 2076** | ‘D. solani’ | *Solanum tuberosum* cv. Nicola | Belgium | 2008 | *fliC*: ‘solani’; qPCR *fliC*: + | + | + |
| **GBBC 2077** | ‘D. solani’ | *Solanum tuberosum* cv. Spunta | Belgium | 2008 | *fliC*: ‘solani’; qPCR *fliC*: + | + | + |
| **GBBC 2078** | ‘D. solani’ | *Solanum tuberosum* cv. Nicola | Belgium | 2008 | *fliC*: ‘solani’; qPCR *fliC*: + | + | + |
| **GBBC 2079** | ‘D. solani’ | *Solanum tuberosum* cv. Nicola | Belgium | 2008 | *fliC*: ‘solani’; qPCR *fliC*: + | + | + |
| **GBBC 2080** | ‘D. solani’ | *Solanum tuberosum* cv. Hermes | Belgium | 2008 | *fliC*: ‘solani’; qPCR *fliC*: + | + | + |
| **LMG 25864,** GBBC 2039 | *D. dianthicola* | *Solanum tuberosum* | Belgium | 2004 | *fliC*: *dianthicola*; qPCR *fliC*: - | - | - |
| **GBBC 2081** | *D. dianthicola* | *Solanum tuberosum* cv. Lady Claire | Belgium | 2008 | *fliC*: *dianthicola*; qPCR *fliC*: - | - | - |
| **GBBC 2082**, PD 2174 | *D. dianthicola* | *Solanum tuberosum* | The Netherlands | 1993 | *fliC*: *dianthicola*; qPCR *fliC*: - | - | - |
| **GBBC 2083**, PD 482 | *D. dianthicola* | *Solanum tuberosum* cv. Ostara | The Netherlands | 1984 | *fliC*: *dianthicola*; qPCR *fliC*: - | - | - |
| **GBBC 2084**, PD 581 | *D. dianthicola* | *Solanum tuberosum* cv. Element | The Netherlands | 1985 | *fliC*: *dianthicola*; qPCR *fliC*: - | - | - |
| **GBBC 2085,** PRI 980 | *D. dianthicola* | *Solanum tuberosum* | The Netherlands |  | *fliC*: *dianthicola*; qPCR *fliC*: - | - | - |

^a^ Multiple strain designations indicates the strains have been deposited in different culture collections.

^b^ . Strains indicated with + were susceptible to infection of the phage, - designated strains were not infected.
